# Metabolic Activation of the Toxic Natural Products From Herbal and Dietary Supplements Leading to Toxicities

**DOI:** 10.3389/fphar.2021.758468

**Published:** 2021-10-20

**Authors:** Yi-Kun Wang, Wen Qun Li, Shuang Xia, Lin Guo, Yan Miao, Bi-Kui Zhang

**Affiliations:** ^1^ Department of Pharmacy, The Second Xiangya Hospital, Central South University, Changsha, China; ^2^ Institute of Clinical Pharmacy, Central South University, Changsha, China

**Keywords:** herbal and dietary supplements-induced liver injury, natural products, metabolic activation, reactive metabolites, toxicity

## Abstract

Currently, herbal and dietary supplements have been widely applied to prevent and treat various diseases. However, the potential toxicities and adverse reactions of herbal and dietary supplements have been increasingly reported, and have gradually attracted widespread attention from clinical pharmacists and physicians. Metabolic activation of specific natural products from herbal and dietary supplements is mediated by hepatic cytochrome P450 or intestinal bacteria, and generates chemical reactive/toxic metabolites that bind to cellular reduced glutathione or macromolecules, and form reactive metabolites-glutathione/protein/DNA adducts, and these protein/DNA adducts can result in toxicities. The present review focuses on the relation between metabolic activation and toxicities of natural products, and provides updated, comprehensive and critical comment on the toxic mechanisms of reactive metabolites. The key inductive role of metabolic activation in toxicity is highlighted, and frequently toxic functional groups of toxic natural products were summarized. The biotransformation of drug cytochrome P450 or intestinal bacteria involved in metabolic activation were clarified, the reactive metabolites-protein adducts were selected as biomarkers for predicting toxicity. And finally, further perspectives between metabolic activation and toxicities of natural products from herbal and dietary supplements are discussed, to provide a reference for the reasonable and safe usage of herbal and dietary supplements.

**GRAPHICAL ABSTRACT:**
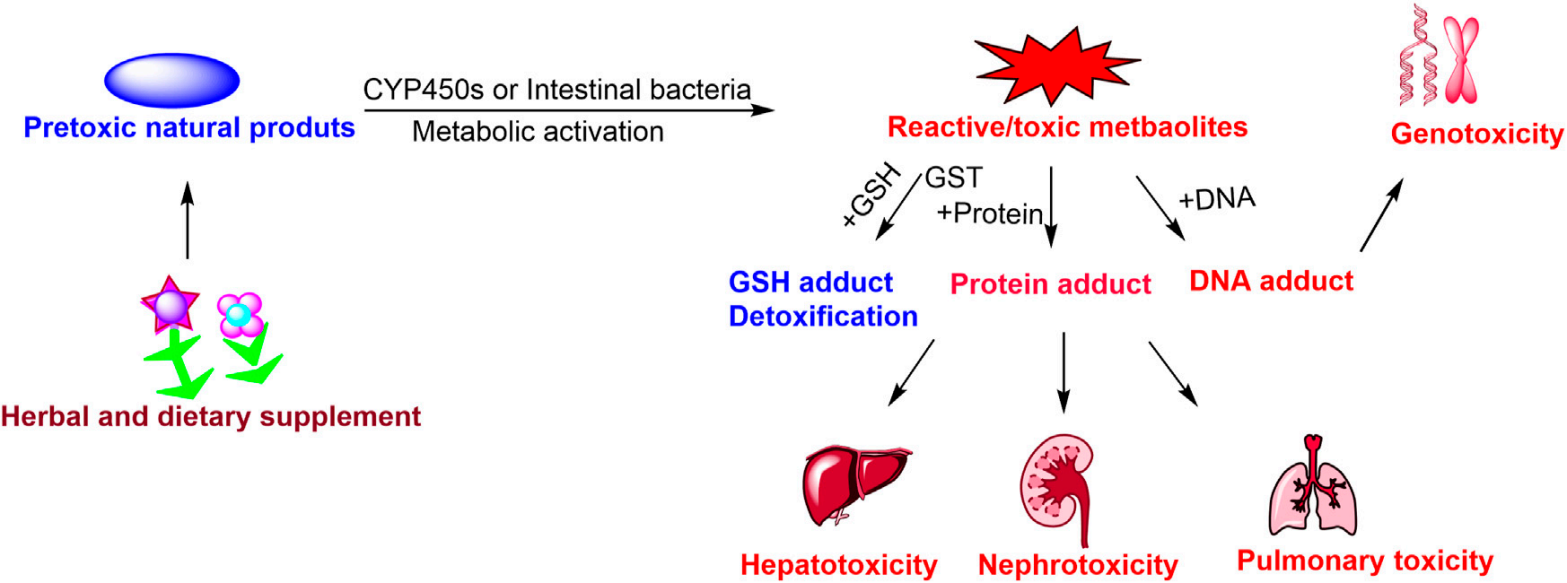


## 1 Introduction

Herbal and dietary supplements are widely used to treat and prevent various diseases for thousands of years worldwide, particularly in Asian countries, including China (Liu et al., 2016), Japan (Watanabe et al., 2011; Hayasaka et al., 2012), North Korea (Jang et al., 2017) and India (Phondani et al., 2010). The usage of herbal and dietary supplements and their preparations or formulation has increased rapidly worldwide over the past three decades. Currently, the curative effects of medicinal plants are obtaining the approval of clinicians in Europe and North American, and the application of herbal remedies has been continuously increasing in complementary and alternative medicine. Approximately 18% of American adults would choose natural drug preparations for treating illnesses (Ekor, 2013). Concomitantly, an increasing number of toxic herbal and dietary supplements are being discovered, reported and verified at the animal and clinical level. Nevertheless, the potential toxicities and adverse reactions greatly limit the reasonable and safe usage of herbal and dietary supplements in clinical.

Hepatotoxicity and nephrotoxicity are two major risk factors to cause synthetic drugs or natural drugs withdraw from the market. Moreover, the prevalence of multiple toxicity caused by herbal and dietary supplements is increasing in the worldwide. The hepatotoxicity (Teschke et al., 2015) and nephrotoxicity (Feng et al., 2018) related to clinical cases caused by traditional Chinese medicine also exist extensively in China, owing to inappropriate use of Chinese medicinal herb (large dosages and/or long-term usage). In addition, herbal and dietary supplements induced liver injury now accounts for 20% of cases of hepatotoxicity in the United States (Navarro et al., 2016). Differ from synthetic drug, natural products from herbal medicines and dietary supplements are more complex and mostly uncertain. The variety of natural products, complexity of multi-ingredient supplements, and unknown concentrations, as well as additional unlabeled substances make the diagnosis and prognostication of toxicity challenging and require further research and attention by the scientific community.

Natural products are widely distributed in herbal and dietary supplements. natural products researcher have isolated and identified a large number of toxic or pre-toxic natural products from medicinal plants or diet (Bunchorntavakul and Reddy, 2012; Posadzki et al., 2013), which usually lead to reversible or irreversible acute organs injury towards animal and humans, and even death. In general, these pre-toxic natural products usually exhibit chemical inertness, and require metabolic activation to form reactive metabolites, which elicit their toxic effects to a certain extent. Extensive research has demonstrated that drug metabolizing enzymes or intestinal bacteria, especially cytochrome P450s (CYP450s), mediate the majority of metabolic activation process, and play a vital catalytic action in formatting reactive intermediates/metabolites. Phase II Enzymes glucuronosyltransferase (UGTs), glutathione S-transferase (GSTs) and sulfotransferases (SULT) mediate the minority of metabolic activation, which closely related to toxicities. Sulfotransferases involve in the metabolic activation of estrogenic (Reinen and Vermeulen, 2015) and aloe-emodin (Li et al., 2019a). UGTs 1A1, 1A9, and 2B7 metabolic activation of rhein responsible for reactive metabolites (Yuan et al., 2016). These reactive intermediates/metabolites are chemically active and electrophilic, if not quenched timely by endogenous nucleophiles, such as antidote glutathione (GSH), or cysteine (Cys). Overdose reactive intermediates/reactive metabolites are also liable to covalently bind to cellular proteins or DNA (Yan et al., 2008), and eventually initiate and trigger a series of toxic effects, including protein abnormal modification, enzyme inactivation, DNA crosslink, formation of immunogenic species, cell death, or oncogene activation, organ injury (Liu et al., 2016), Therefore, metabolic activations is the critical initiating factor responsible for toxicity, and these intermediates/reactive metabolites are usually toxic metabolites of natural products.

The present review focuses on the toxicity of natural products, analyzing the key role of metabolic activation in their toxicities. According to the different catalysis of metabolic activation, they are divided into CYP450s mediated metabolic activation and intestinal bacteria mediated metabolic activation. Moreover, CYP450s mediated metabolic activation are categorized into three categories, pyrrolizidine alkaloids, furan derivatives, epoxy diterpenoids, anthraquinones aristolochic acids, bisbenzylisoquinoline, alkenylbenzenes, based on the types of potentially toxic natural products.

## 2 CYP450s Mediated Metabolic Activation of Natural Products Leading to Toxicity

### 2.1 Metabolic Activation of Pyrrolizidine Alkaloids Leading to Hepatotoxicity, Phototoxicity and Pulmonary Toxicity

Pyrrolizidine alkaloids (PAs) are the class of common toxic natural products widely distributed in over 600 herbals around the world, and are found in approximately 3% of the world’s flowering plants (Robertson and Stevens, 2017). Multiple PAs mainly exist in species of the plant families *Boraginaceae* (all genera), Compositae (tribes Senecionae and Eupatoriae) and Leguminosae (genus *Crotalaria*) (Watanabe et al., 2011), and they are also could be isolated from multiple medicinal plants, including *crotalaria mucronata, crotalaria sessiiflora, senecio scandens, gynura japonica, tussilago farfara, eupatorium japonicum, heliotropium europaeum, eupatorium fortunei, arnebia euchroma, tephroseris kirilowii, lithospermum erythrorhizon.* They are also frequently applied in clinical practice of traditional Chinese medicine (Tang and Hattori, 2011). Moreover, consumers can be easily exposed to these hepatotoxic PAs through consumption of PAs contained in herbal and dietary supplements, for instance, herbal tea (Merz and Schrenk, 2016), and dietary components such as milk and honey (Griffin et al., 2015). Currently, PAs-caused hepatic damage has become a world-wide problem of drugs and food safety, and seriously threatened people’s health.

**FIGURE 1:**
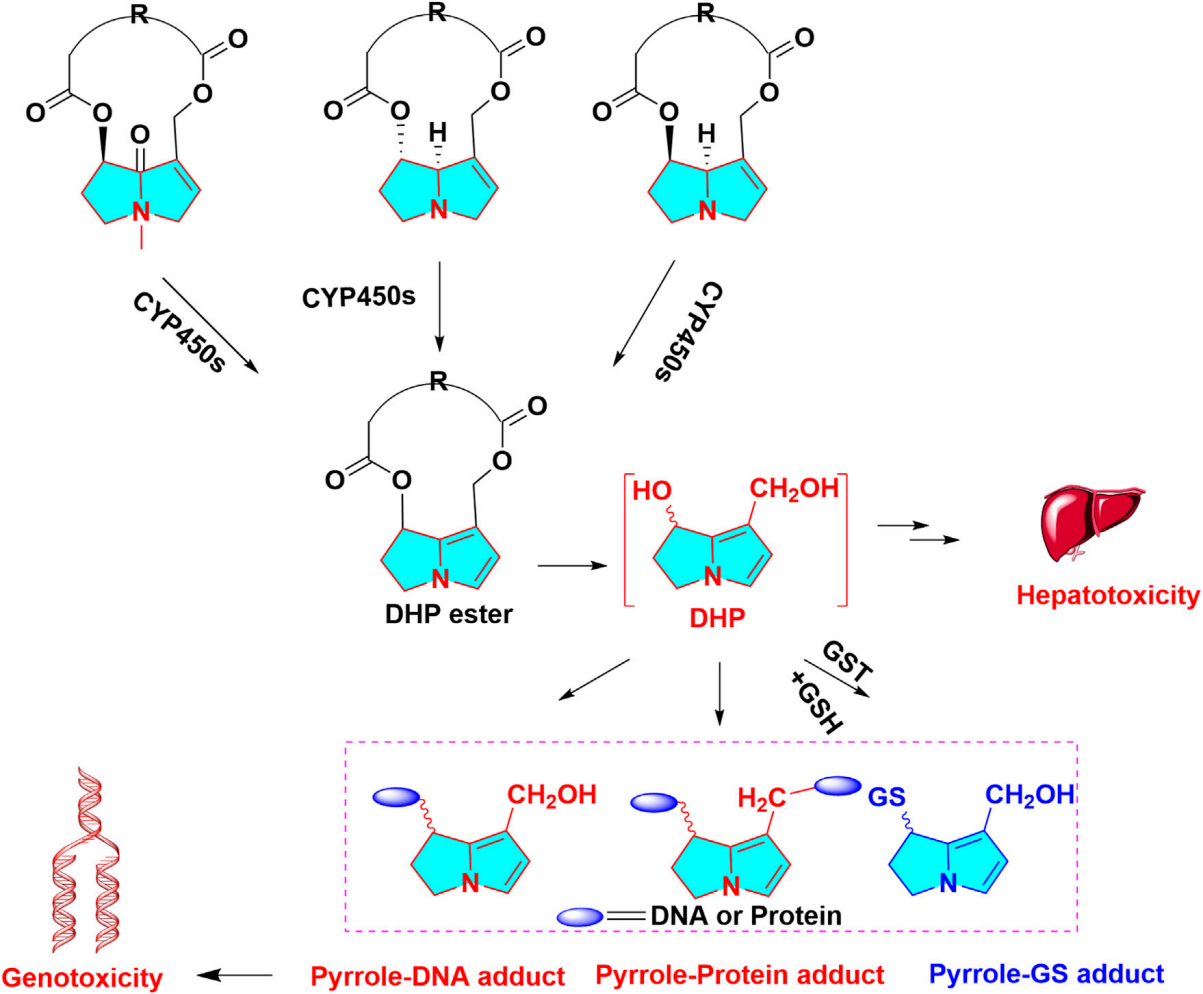
Metabolic activation in hepatotoxicity of PAs, otonecine-type PA, heliotridine-type PA and retronecine-type PA are oxidized to dehydro PAs, then dehydro PAs are hydrolyzed to DHP, reactive metabolites can capture GSH, protein, DNA, forming GSH, protein, DNA adduct, respectively.

More than 600 PAs and PA *N-*oxide derivatives have been isolated from medicinal plants at present, and the great majority of them exhibit obvious hepatic damage towards human and animal. The PAs with an α,β-unsaturated necine skeleton in the structure can exert multiple toxic effects, such as hepatotoxicity (Neuman et al., 2015), genotoxicity (Fu et al., 2004), cytotoxicity (Forsch et al., 2017), phototoxicity, and photogenotoxicity (Wang et al., 2014a). Therefore, the unsaturated necine base is toxic functional group of hepatotoxic PAs. PAs exert hepatotoxicity through metabolic activation by hepatic CYP450s to generate reactive intermediates. Unsaturated necine core type of PAs with a double bond in the base can show potent hepatotoxicity, owing to metabolic activation (Ruan et al., 2014a), whereas these natural products with saturated necine moiety does not cause liver damage (Ruan et al., 2014b). The formation of hepatotoxicity was assigned to the metabolic activation of PAs in the liver and generated the reactively toxic metabolites. Metabolic activation of PAs needs three steps: 1) Oxidation (dehydrogenation or aromatization) of otonecine-type, heliotricline-type and retronecine-type, then produces reactive intermediates of pyrrolic esters (didehydro-pyrrolizidine, DHP esters) mediated by CYP450s. Particularly, CYP3A4 can catalyze the oxidation reaction of PAs to form toxic metabolites. Molecular docking also simulated the role of CYP3A4, which displayed that the C3 of lasiocarpine and retrorsine and C26 of senkirkin were closest to the catalytic heme Fe (CYP3A4 active site); 2) Hydrolysis of DHP esters forms didehydro-pyrrolizidine (DHP) (necines and necic acids); 3) *N-*oxidation of PAs can produce PAs *N-*oxides. The formation of reactive pyrrolic intermediates (DHP, metabolic pyrrole) has been commonly regarded as the critical step for PAs-caused toxicities (Figure 1). Once formed, DHP tends to capture intracellular nucleophilic biomolecules rapidly, such as capture reaction with GSH to form GSH conjugates (Chen et al., 2016), or attack macromolecules DNA or proteins to form pyrrole-DNA conjugates (Xia et al., 2015) and pyrrole-protein adducts. It is generally accepted that most GSH addition reaction are a self-defending action against toxicities of PAs. Moreover, this reactive DHP can irreversibly bind to hepatic proteins, resulting in hepatotoxicity. Pyrrole-protein adducts were reported to have potential value as the non-invasive biomarkers of PAs -induced hepatotoxicity (Xia et al., 2016). Pyrrole-hemoglobin adducts, a more feasible potential biomarker of pyrrolizidine alkaloid exposure, was discovered (Ma et al., 2019).

Apart from hepatotoxicity, approximately half of the 660 PAs and PA *N*-oxides that have been characterized are cytotoxic (Forsch et al., 2017), genotoxic (Fu et al., 2004), tumorigenic (Neuman et al., 2015; Fu, 2017), phototoxic and photogenotoxic (Wang et al., 2014a). Metabolic activation pathways of PAs can lead to obvious liver tumor initiation. PAs can induce DNA adduction, DNA damage, and activation of tumorigenic hepatic progenitor cells, which initiate hepatocarcinogenesis. Pyrrolizidine alkaloid-protein adducts were detected in 32% of surgically resected specimens from 34 patients with liver cancer in Hong Kong (He et al., 2021a). Hepatic cytochrome P450s mediated metabolic activation induced acute pulmonary injury (He et al., 2021b).

### 2.2 Metabolic Activation of Furan Derivatives-Induced Hepatotoxicity

Furan derivatives are important aromatic heterocyclic compounds. Early reports indicated that natural furan derivatives were present in numerous processed food, with the highest contents found in coffee. *Teucrium chamaedrys*, a traditional food and medicinal plant, rich in furan derivatives have been reported to exhibit multiple toxicities, especially for liver injury (Lekehal et al., 1996; Gori et al., 2011; Nencini et al., 2014)**.** As potentially hepatotoxic natural products, furanoterpenoids are widely distributed in food, beverages, medicinal plants, and even marine organisms. Multiple furanoterpenoids, can be classified into three types of furanomonoterpenes, furanoditerpenes and furanotriterpenes (Huang et al., 2020). Reported hepatotoxic furanomonoterpenes majorly included 4-ipomeanol (Parkinson et al., 2016), (-)-ngaione (Ng, 1983), teucrin A, teuchamaedryn A (Lekehal et al., 1996), menthofuran (Lassila et al., 2016). Reported hepatotoxic furanoditerpenes contained teucrin A, teuchamaedryn A (Lekehal et al., 1996), diosbulbin D and diosbulbin E, columbin. Reported hepatotoxic furanotriterpenes contained toosendanin (Yu et al., 2014), rutaevin (Liu et al., 2020), and nomilin (Zhang et al., 2020a), distributed in Meliaceae medicinal plants.

As a medicinal plant, the rhizome of *Dioscorea bulbifera*, has been extensively used to treat tumors and struma in East Asia (Yonemitsu et al., 1993). Numerous clinical cases of liver injury have been reported after administration of *D. bulbifera* and its formulation (Jiang et al., 2004). Animal studies also revealed that oral administration of ethanol extracts of *D. bulbifera* could cause the significant liver injury, together with increased lipid peroxide levels in liver (Wang et al., 2010). Belonging to clane-type diterpene lactone with furane ring, diosbulbin B, diosbulbin D, and 8-epidiosbulbin E Acetate are principal constituents of *D. bulbifera*, which were verified to cause obvious liver toxicity towards on rat or mouse, respectively (Lin et al., 2015; Lin et al., 2016a; Lin et al., 2016b). Although there is no furan in the structure of pulegone, it can be initially biotransformed to menthofuran after metabolism (Thomassen et al., 1990), and menthofuran can further generate toxic metabolites, after metabolic activation (Ravindranath et al., 1984; Ravindranath et al., 1986). Generally, most furan derivatives are usually hepatoxic and/or carcinogenic. Metabolic activation of these pre-toxic furan derivatives is initially biotransformed by epoxidation, and generate a cis-enedione intermediate. These reactive intermediates can attack cellular nucleophiles (protein or DNA) to trigger toxicities, further leading to hepatotoxicity (Peterson, 2013). Diosbulbin B was elected as a representative case in this section to illustrate the role of metabolic activation. Diosbulbin B can cause obvious liver jury, while the furan ring of diosbulbin B was chemically reduced to a tetrahydrofuran moiety, no obvious liver damage was observed in animals after administration of tetrahydro-diosbulbin B. Therefore, the structure-toxicities relationship of furanoterpenoids revealed that unsaturated furan ring moiety is the hepatoxic functional group (Table 1). Meanwhile, CYP450s-mediated epoxidation occurred in the furan of diosbulbin B was regarded as metabolic activation, forming a reactive intermediate of cis-enedial (Yang et al., 2014; Lin et al., 2016a). The formation of cis-enedial intermediate in liver microsomes (Lin et al., 2014), and CYP3A4-transfected primary rat hepatocytes, HepG2 and L02 cells (Jiang et al., 2017) were significantly inhibited by the potent CYP3A inhibitor of ketoconazole (Lin et al., 2014; Jiang et al., 2017).

**TABLE 1:** Metabolic activation of natural products in their toxicities.

Parents	Class	Herbal source	Reactive metabolites	Involved enzymes	Toxicities	References
Diosbulbin B	Furanoterpenoids	*Dioscorea bulbifera*	cis-Enedial	CYP3A4	Liver injury	Lin et al. (2014) Lin et al. (2016a)
8-Epidiosbulbin E acetate	Furanoterpenoids	*Dioscorea bulbifera*	cis-Enedial	CYP3A4	Liver injury	Lin et al. (2015); Lin et al. (2016b)
Diosbulbin B	Furanoterpenoids	*Dioscorea bulbifera*	cis-Enedial	CYP3A4	DNA Adduction	Lin et al. (2019)
8-Epidiosbulbin E acetate	Furanoterpenoids	*Dioscorea bulbifera*	cis-Enedial	CYP3A4	DNA Adduction	Lin et al. (2019)
4-Ipomeanol	Furanoterpenoids	*Ceratocystis fimbriata* Ellis	cis-Enedial	CYP450s monooxygenases	Pulmonary toxin	Buckpitt and Boyd, (1982)
4-Ipomeanol	Furanoterpenoids	*Ceratocystis fimbriata* Ellis	cis-Enedial	CYP4B	Pulmonary toxin	Parkinson et al. (2012)
4-Ipomeanol	Furanoterpenoids	*Ceratocystis fimbriata* Ellis	Enedial intermediate	CYP4B1	Pulmonary toxin	Baer et al. (2005); Parkinson et al. (2013); Verschoyle et al. (1993)
Teucrin A	Furanoterpenoids	*Teucrium chamaedrys*	Enedial	CYP450s	Liver injury	Druckova and Marnett, (2006)
Teuchamaedryn A	Furanoterpenoids	*Teuchrium chamaedrys*	Enedial	CYP450s	Liver injury	Lekehal et al. (1996)
Pulegone	Furanoterpenoids	*Mentha haplocalyx*	Menthofuran	CYP450s		
Toosendanin	Furanoterpenoids	MeLia toosendan Sieb	Enedial intermediate	CYP 3A4	Liver injury	Yu et al. (2014)
Emodin	Anthraquinones	Polygoni multiflori	Quinone intermediates	CYP 3A	Hepatotoxicity	Jiang et al. (2018)
Aloe-Emodin	Anthraquinones	*Polygoni multiflori*	Quinone intermediates	Sulfotransferases	Cytotoxicity	Li et al. (2019a)
Physcion	Anthraquinones	*Polygoni multiflori*	Quinone intermediates	CYP450s	Hepatotoxicity	Qin et al. (2018)
Rhein	Anthraquinones	*Polygoni multiflori*	Quinone intermediates	CYP 2C9	Hepatotoxicity	Xu et al. (2018)
Chrysophanol	Anthraquinones	*Polygoni multiflori*	Quinone intermediates	CYP 1A2	Hepatotoxicity	Sun et al. (2018)
Lucidin-3-O-primiveroside	Anthraquinones	*Rubia tinctorium Linn*	Lucidin		DNA adduct or DNA lesion	Yockey et al. (2017)
Dauricine	Bisbenzylisoquinolines	*Menispermum dauricum*	Quinone methide intermediate	CYP 3A	Pulmonary toxicity	Jin et al. (2010); Jin et al. (2012)
Berbamine	Bisbenzylisoquinolines	Berberis amurensis	Quinone methide intermediate	CYP 3A4	Pulmonary toxicity	Sun et al. (2017); Sun et al. (2017)
Tetrandrine	Bisbenzylisoquinolines	Stephania tetrandra	Quinone methide intermediate	CYP450s	Pulmonary toxicity	Jin et al. (2011); Tian et al. (2016)
Tetrandrine	Bisbenzylisoquinolines	Stephania tetrandra	Quinone methide intermediate	CYP 3A5	Pulmonary toxicity	Tian et al. (2016)
Neferine	Bisbenzylisoquinolines	Nelumbo nucifera	Quinone methide intermediate	CYP3A4	Pulmonary toxicity	Shen et al. (2014)
3-Methylindole	Bisbenzylisoquinolines		3-Epoxy-3-methylindoline	CYP450s	Pulmonary disease	Skordos et al. (1998)
3-Methylindole	Bisbenzylisoquinolines		Reactive iminium	CYP450s	Pulmonary disease	Yost (1989); Huijzer et al. (1987)
Estragole	Alkenylbenzenes	Tarragon, sweet basil and sweet fennel	1′-Sulfooxyestragole	CYP 1A2, 2A6	Hepatocellular carcinomas	Jeurissen et al. (2007)
Safrole	Alkenylbenzenes	betel oil, sassafras oils, and camphor oil	1′-Sulfooxysafrole	CYP 2C9, 2A6, 2D6,2E1	Hepatocellular carcinomas	Jeurissen et al. (2004)
Methyleugenol	Alkenylbenzenes	*Acacia senegal*., *Cinnamomum verum*	1′-Sulfooxymethyleugenol	CYP 1A2, 2C9,2C19	Hepatocellular carcinomas	Jeurissen et al. (2006)
Apiol	Alkenylbenzenes	Parsley	1′-Sulfooxyapiol	CYP450s	DNA RNA adduct	Alajlouni et al. (2016)
Myristicin	Alkenylbenzenes	*Myristica fragrans*	1′-Hydroxymyristicin	CYP1A1	Cytotoxicity in HepG2	Zhu et al. (2019)
Quercetin	Flavonoids	Quercus Linn	Quinone and quinone methides	Liver extract	Mutagenicity	Vrijsen et al. (1990)
Fisetin	Flavonoids	Genus *Citrus*	Geraldol	Catechol-O-methyltransferase (COMT)	Cell cycle arrest	Poór et al. (2016)
Quercetin	Flavonoids	*Quercus dentata*	Isorhamnetin		Cell cycle arrest	Poór et al. (2016)
Fisetin	Flavonoids	Genus *Citrus*	Geraldol	Catechol-O-methyltransferase (COMT)	Cell cycle arrest	Poór et al. (2016)
Quercetin	Flavonoids	*Quercus dentata*	Isorhamnetin		Cell cycle arrest	Poór et al. (2016)
Kaempferol	Flavonoids	*Kaempferia rotunda*		Hepatic S9 microsomal fraction	Cytotoxicity	Soares et al. (2006)
Apigenin	Flavonoids	*Apium graveolens*	Luteolin	CYP 1A1,B1	Antiproliferative activity in breast cancer cells	Wilsher et al. (2017)
Tangeretin	Flavonoids	Genus *Citrus*	4′- OH-tangeretin	CYP 1A1,1B1	Antiproliferative activity in breast cancer cells	Surichan et al. (2018a)
Nobiletin	Flavonoids	Genus *Citrus*	NP1	CYP 1A1,1B1	Antiproliferative activity in breast cancer cells	Surichan et al. (2018b)
Nobiletin	Flavonoids	Genus *Citrus*	Demethylated nobiletin	CYP 1A1,1B1	Antiproliferative activity in breast cancer cells	Surichan et al. (2012)
Eupatorin	Flavonoids	*Eupatorium fortunei*	Unidentified metabolites	CYP 1A1,1B1	Antiproliferative activity in breast cancer cells	Androutsopoulos et al. (2008)
Diosmetin	Flavonoids	*Spermadictyon suaveolens*	Luteolin	CYP1	Antiproliferative activity in breast cancer cells	Androutsopoulos et al. (2009)
Luteolin	Flavonoids		ortho‐Benzoquinone metabolite	CYP450s	Cytotoxicity	Shi et al. (2015)
Arbutin	Iridoids	Arctostaphylos uva-ursi	Hydroquinone	Rat intestinal flora	Cytotoxicity	Kang et al. (2011)
Geniposide	Iridoids	Gardenia jasminoides Ellis	Genipin	Intestinal flora	Cytotoxicity in HepG2 cells	Kang et al. (2012); Li et al. (2019b)
Cycasin	Alkaloids	Cycas revoluta Thunb	Methyl azoxymethanol	Glycosylases	mutagenic	Morgan and Hoffmann, (1983)
Daidzin	Flavonoids	Glycine max	Daidzein, calycosin	Human Faecal suspension	Cytotoxicity in tumour cell lines	Kim et al. (1998)

**FIGURE 2:**
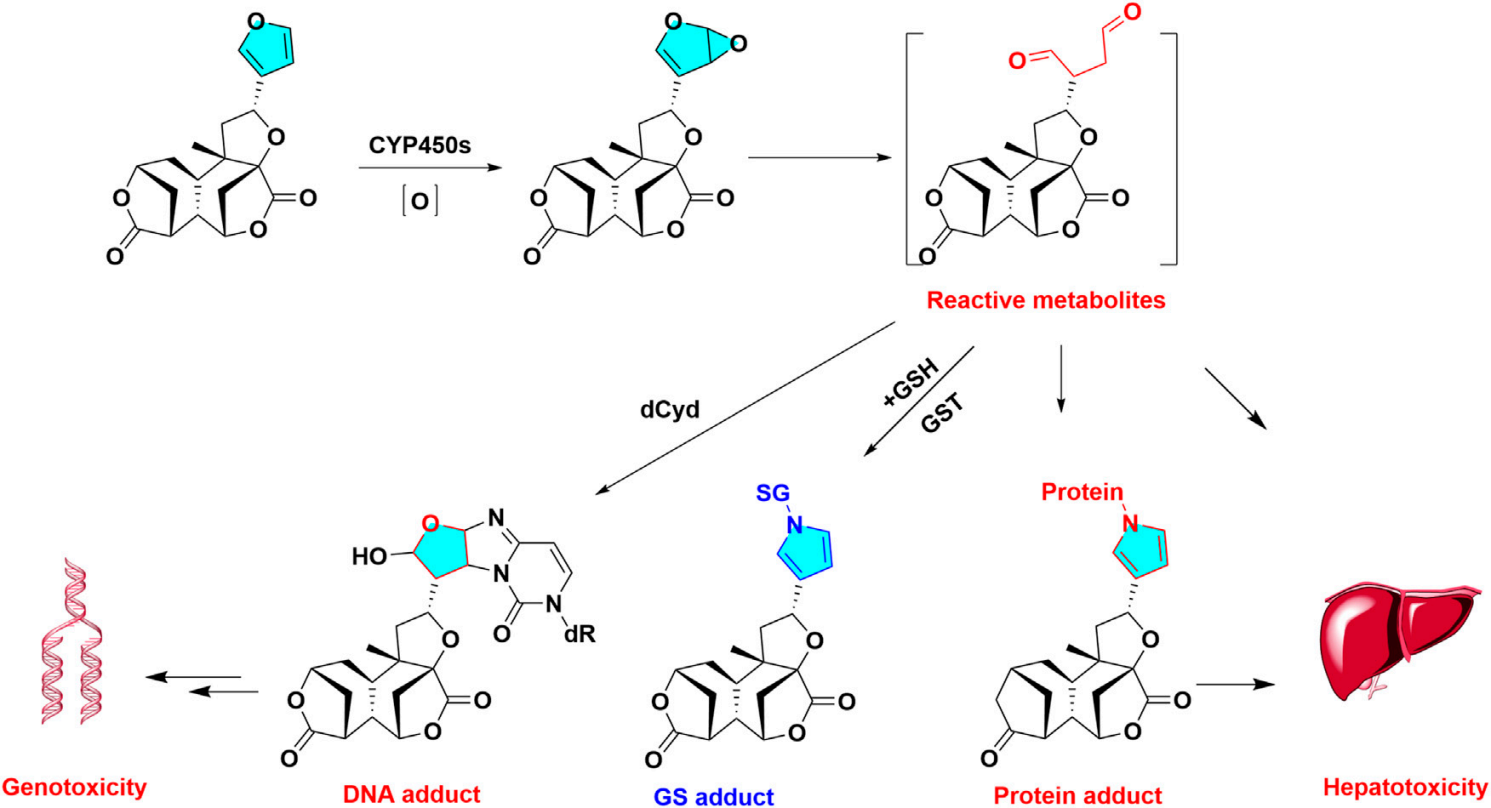
Metabolic activation diosbulbin B induced hepatotoxicity. Diosbulbin B is epioxidized and hydrolyzed to reactive metabolites of cis-Enedial, reactive metabolites can capture GSH, protein, DNA, forming GSH, protein, DNA adduct, respectively.

Further study disclosed the cis-enedial intermediate is substantially nucleophilic, which was liable to be trapped by *N-*acetyl lysine, *N-*acetyl cysteine (NAC) or GSH in rat and human liver. The covalent bind with free glutamyl-amine of GSH is conducted in Schiff-base manner to form N-linked conjugates. The exhaustion of hepatic GSH was also observed in diosbulbin B treated animals (Lin et al., 2014) (Figure 2). The sensitive mass spectrometry strategy has been built to detect furans reactive metabolites plus GSH conjugates, such as neutral loss scanning of 290.0573 Da in the positive ionization mode, and precursor ion scanning of *m/z* 143.0462^+^ in the negative ionization mode (Wang et al., 2014b). Moreover, cis-enedial-protein adduct can be analyzed, cysteine (Cys) and lysine (Lys) residue of protein could be easily captured by cis-enedial to form three kinds of protein abnormal modification, through Cys adduction, Schiff’s base, or Cys/Lys crosslink (Wang et al., 2017a), respectively. In addition, the protein adductions of reactive metabolites of furans were determinated as Cys-and Lys-based protein adductions with the reactive metabolites (Wang et al., 2015). Reactive metabolite of Teucrin A-protein adduct was used to identification of the protein targets of the Teucrin A, the protein targets was origin from mitochondrial and endoplasmic reticulum origin (Druckova et al., 2007).

Apart from hepatotoxicity, furan has been classified as “possibly carcinogenic to human” by IARC, a great concern has been addressed to the detection of this substance naturally-occurring in food. Metabolic activation of diosbulbin B and 8-epidiosbulbin E acetate resulted in DNA adduction. In addition, furan ring is an essential toxic structural alert responsible for furan alkaloids of dictamnine which can cause hepatotoxicity, and epoxy of furan ring was also discovered in the metabolism of dictamnine. Furoquinoline alkaloid dictamnine, a furoquinoline alkaloid of the Rutaceae plant family, can resulted in carcinogenicity, cytotoxicity, and genotoxicity via CYP450s mediated metabolic activation. Moreover, a variety of furanocoumarins, including 8-methoxypsoralen and other furanocoumarins, can cause mechanism-based inactivation of CYP 450 (Koenigs and Trager, 1998).

### 2.3 Metabolic Activation of Epoxy Diterpenoids-Induced Hepatotoxicity

Belonging to the Celastraceae family, *Tripterygium wilfordii* Hook has been used for numerous centuries in traditional Chinese medicines for treatment of rheumatoid arthritis (Tao et al., 2002), immune complex nephritis and systemic lupus erythematosus (Kao et al., 2010). Previous study revealed the total epoxy diterpenes of *T. wilfordii* exhibited significant liver injury in clinical (Chai et al., 2011). Triptolide (TP) is an abietane type diterpene with triepoxy ring, one of many toxic ingredients of *T. wilfordii*. Although TP is a promising lead compound for treatment of rheumatoid arthritis, cancer, and erythema atrophicans, its clinical efficacy and safety are greatly limited by its obvious multiple toxicity, including hepatotoxicity and nephrotoxicity (Wang et al., 2017b).

Epoxy group are a toxic functional group. Owing to triepoxy ring, triptolide (parents) exhibit chemically reactive activity. The biotransformation of triptolide were hydrolysis and hydroxylation reactions *in vivo* metabolism (Peng et al., 2012; Liu et al., 2013). Total 8 NAC metabolites of triptolide were observed in rat urine. The formation of NAC or GSH conjugates indicated the metabolic activation of epoxy may occur in the metabolic metabolism (Du et al., 2011).The hepatotoxicity of triptolide can be affected by multiple factors, particularly drug metabolic enzymes and transporters. Previous report demonstrated that CYP450s-mediated metabolic activation played the key role in triptolide-induced hepatic damage towards rat hepatocyte (Zhuang et al., 2013). Triptolide induced liver injury could be attenuated by CYP450s broad spectrum inhibitor, 1-aminobenzotriazole (Zhuang et al., 2013). In addition, knockout of hepatic CYP 450 reductase could exacerbate triptolide -induced toxicity in mice (Xue et al., 2011), while pretreatment with CYP3A inducer of dexamethasone could protect against triptolide originated liver injury in rat (Ye et al., 2010), suggested CYP 450s mediated the metabolic activation of triptolide, leading to hepatotoxicity.

Metabolic epoxidation is a critical step in the progress of specifying xenobiotics-induced hepatotoxicity (Wang et al., 2017c). Triptolide possesses multiple obvious potent toxicities, which may be related to the bioactivation of structural alerts (triepoxy) in the metabolism. Owing to the remarkable toxicity, herbal prescriptions containing *T. wilfordii* or *T. hypoglaucum* should be carefully administered in clinical.

### 2.4 Metabolic Activation of Anthraquinones-Induced Hepatotoxicity

Anthraquinones are a class of functionally diverse natural products structurally related to anthracene, which widely exist in Polygonaceae medicinal plants, including *Fallopia multiflora*, *Rheum palmatum* and *Aloe vera*, these medicinal plants are massively consumed in the world. Anthracene possesses significant liver injury, anthraquinone is chemically similar to anthracene, there is a potential risk of hepatotoxicity underlie the application of anthraquinones. These anthraquinones may generate reactive metabolites in the metabolic activation process. The toxic anthraquinones and their metabolic activation are clearly shown in Table 1.

*Polygoni multiflori* radix is a popular medicinal plant, extensively used in China, Japan and Korea, which show anti-aging effect and health care value. However, *polygoni multiflora* and their total anthraquinones were reported to cause hepatotoxicity clinically (Furukawa et al., 2010; Kyoung Ah et al., 2011). Emodin is a common anthraquinone component widely distributed in Polygonaceae plants, such as *polygoni multiflori* and *polygonum multiflorum*. Three phase I metabolites of emodin, including 2-hydroxyemodin, 5-hydroxyemodin, and ω-hydroxyemodin, were observed in CYP1A2 and CYP2C19 incubations. Three hydroxylated metabolites of emodin were found to be electrophilic species, reactive to NAC and GSH (Qin et al., 2016). Another anthraquinone compound, physcion, and its oxidative metabolites were also reported to be conjugated with NAC and GSH after metabolic activation (Qin et al., 2018). Based on structure-toxicity relationship analysis, the para-quinone was confirmed as toxic moiety in the structure of anthraquinones. CYP450s mediated metabolic activation was closely associated with the toxicity of anthraquinones.

**FIGURE 3:**
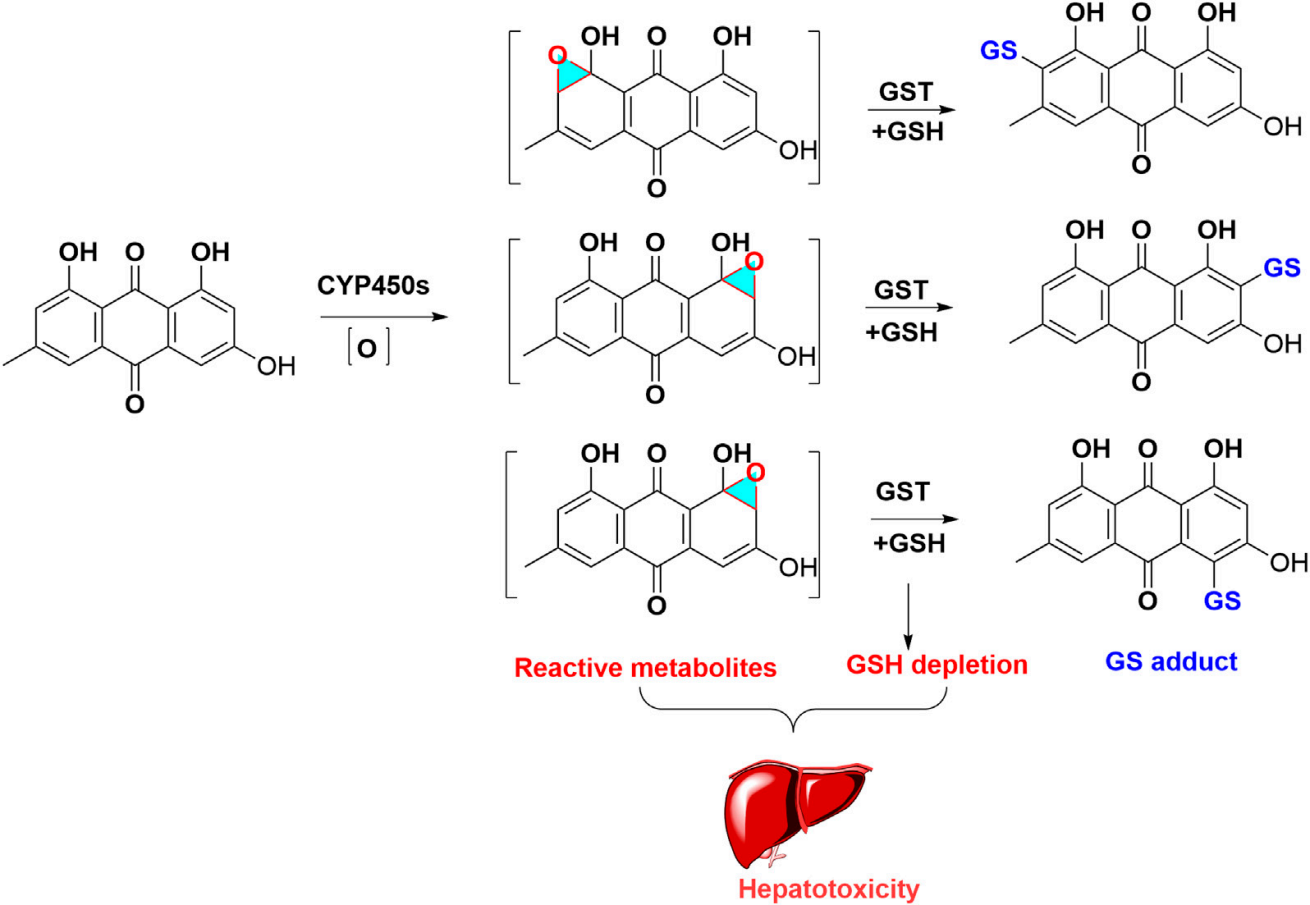
Metabolic activation of emodin induced hepatotoxicity. Emodin formed oxidated intermediates metabolites, these reactive metabolites captured with GSH, and produced GSH adduct, which led to GSH exhaustion.

*Para*-Quinone was characterized as toxic functional group and structural alert of anthraquinones. Owing to the chemically reactive activity of *para*-quinone, epoxidation firstly occurs in the metabolism of anthraquinones and forms reactive metabolites, which can further conjugate with nucleophilic substance, including NAC and GSH. The depletion of GSH would aggravate the anthraquinones induced hepatotoxicity (Figure 3). Currently, the related data about mechanism of anthraquinones-caused hepatotoxicity and nephrotoxicity is limited, which need further research.

### 2.5 Metabolic Activation of Bisbenzylisoquinoline-Induced Pulmonary Toxicity

Bisbenzylisoquinoline alkaloids (BBI) are a large kind of natural product, which usually consist of two benzylisoquinoline moieties by the linker of carbon–carbon bridge or ether bridge in their structure. This class of alkaloids extensively distributed in multiple herbs, majorly existed in plants of Annonaceae, Berberidaceae, Menispermaceae, Ranunculaceae, and Magnoliaceae family (Gao and Xiao, 1998). Numerous medicinal plants and diets were reported to contain this type alkaloids, which have been used as traditional medicines in East Asia, such as *Menispermum dauricum, Mahonia fortune, Stephania japonica, Stephaniae tetrandrae* and so on*.* Reported pharmacological activities of bis-benzylisoquinoline alkaloids majorly including antimalarial, anti-inflammatory, anticancer, immunosuppression and anti-hepatitis activities (Gao and Xiao, 1998)^.^

**FIGURE 4:**
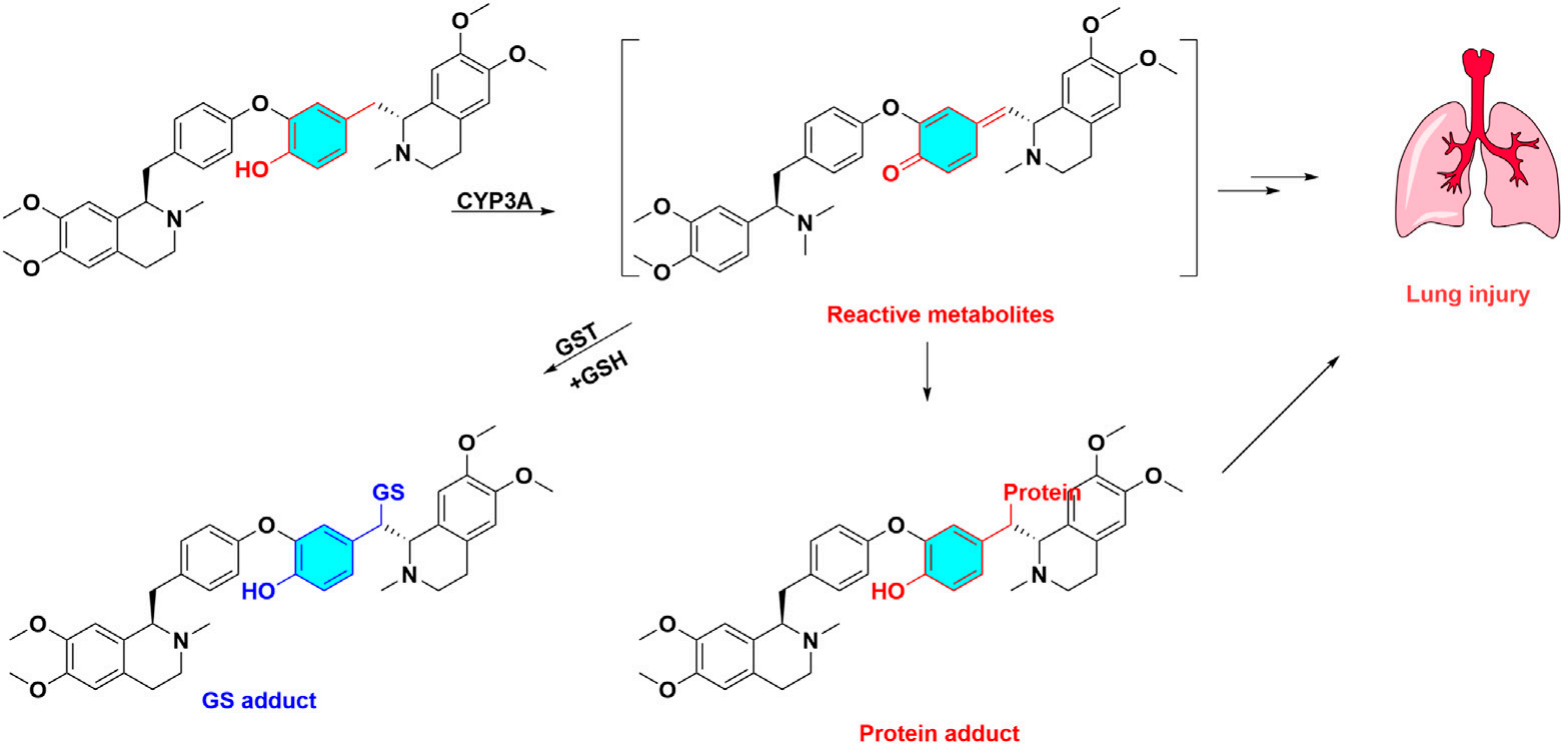
Metabolic activation and toxicity of dauricine. Dauricine are biotransformed quinone methide intermediate catalyzed by CYP3A, these reactive metabolites capture GSH and protein, then produce GSH adduct, protein adduct, at last led to lung injury.

Numerous toxic bisbenzylisoquinoline alkaloids and their metabolic activation are summarized in Table 1. As a typical case, dauricine is discussed in this section to illustrate the role of metabolic activation. Dauricine, a bisbenzylisoquinoline alkaloid, is the major bioactive component of *Menispermum dauricum*. As regards to its metabolism, four GSH conjugates of dauricine were detected in rat bile or HLMs incubations with supplemented NADPH and GSH. The reacted sites of GSH addition were elucidated as 6-position of phenol moiety, demonstrating that metabolic activation occurred in phenol ring, which generated reactive quinone intermediates via oxidation. Recombinant human CYP450 enzymes revealed CYP3A4 was the major metabolic enzyme responsible for the bioactivation of dauricine (Wang et al., 2008). Moreover, a reactive quinone methide metabolite of dauricine was observed and identified in MLMs, which spontaneously captured by GSH (Figure 4), and its GSH adducts can be suppressed by CYP3A inhibitor ketoconazole (Jin et al., 2010). Moreover, ketoconazole could counteract the increased lactate dehydrogenase activity induced by dauricine (Jin et al., 2010), and reverse pulmonary cellular GSH depletion and cell apoptosis in the pulmonary injury caused by dauricine (Jin et al., 2012). The GSH depletory agent of l-buthionine sulfoximine showed potentiating effect on cytotoxicity and apoptosis caused by dauricine (Jin et al., 2012), demonstrating the pulmonary toxicity was associated with the CYP3A mediated metabolic activation. The reactive quinone methide intermediate of dauricine can covalently medicated protein, and form quinone methide-protein adduct, which has been detected by liquid chromatography-mass spectrometry (LC-MS/MS) (Xie et al., 2016).

Apart from bisbenzylisoquinoline alkaloids, toxic bisbenzylisoquinolines also included berbamine, tetrandrine and neferine (Table 1). The quinone methide intermediate of berbamine *in vitro* and *in vivo* metabolism were detected by LC-MS/MS, which can be covalently bind with NAC to generate NAC-derived adducts. CYP3A4 also played a key role in the metabolic activation process of berbamine (Sun et al., 2017). Metabolic activation of tetrandrine can lead to pulmonary toxicity toward CD-1 mice (Jin et al., 2011), and CYP3A5 mediated bioactivation was also closely associated with cytotoxicity of tetrandrine (Tian et al., 2016). CYP3A4 predominantly catalyzed the formation of neferine-GSH conjugates, and GSH depletion significantly aggravated neferine-induced cytotoxicity (Shen et al., 2014). Taken together, *para-*methylene phenol is key toxic structural alerts of bisbenzylisoquinoline alkaloids*,* and activated *para*-quinone methides bio transforming from *para -*methylene phenol is a pivotal step.

### 2 6 Metabolic Activation of Alkenylbenzenes-Induced Carcinogenicity

Dietary alkenylbenzenes are a class of aromatic natural products, which are presented in diversified vegetables, spices and medicinal plants, such as cinnamon, clove nutmeg, pepper, fennel, anise and basil. The common natural alkenylbenzenes majorly include estragole, safrole, methyleugenol, elemicin and myristicin. There are substantial evidences supporting for the genotoxicity and carcinogenicity of allylalkoxybenzenes (Rietjens et al., 2014).

**FIGURE 5:**
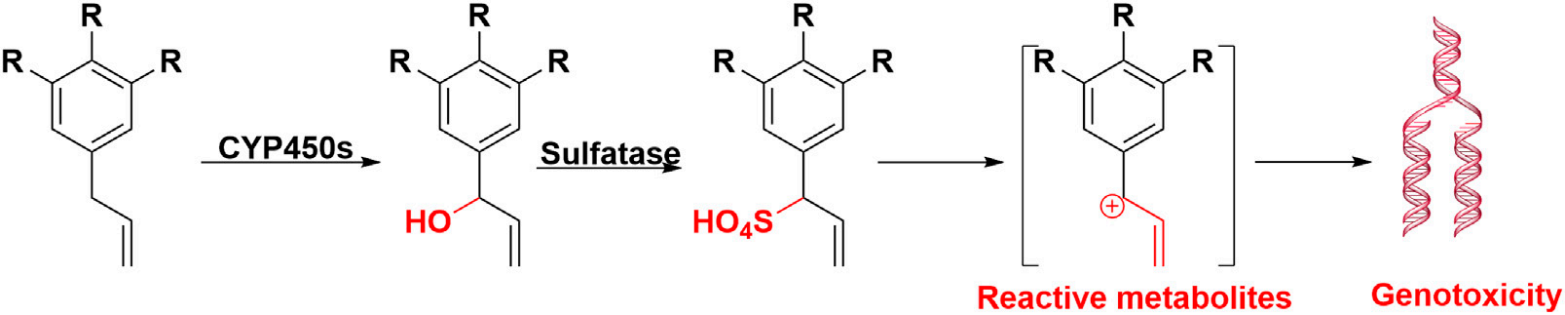
Metabolic activation of alkenylbenzenes induced carcinogenicity. After hydroxylation and solidification, alkenylbenzenes were formed carbocation intermediate, at last produced DNA adducts, and led to carcinogenicity.

The genotoxic alkenylbenzenes and their metabolic activation are listed in Table 1. The initial toxic metabolites of alkenylbenzenes were their 1′-hydroxy derivatives, such as 1′-hydroxysafrole, and their ultimate carcinogenic metabolites are their 1′-sulfooxy derivatives, such as 1′-sulfooxysafrole (Borchert et al., 1973; Boberg et al., 1983), which can be degraded to alkylating carbocations intermediates. These intermediates are electrophilic and reactive, which can conjugate DNA, leading to genotoxicity and carcinogenicity. Therefore, 1′-sulfooxyalkenylbenzenes were elucidated as tumor-initiating metabolites. Overall, the bioactivation of alkenylbenzenes underwent three steps: 1) Hydroxylation reaction at the alkene side chain 1′ site, alkenylbenzenes can be transformed into 1′-hydorxyalkenylbenzenes, therefore, allyl is toxic functional group of alkenylbenzenes; 2) Sulfation reaction, 1′-hydroxyalkenylbenzenes can be sulfated into 1′-sulfooxyalkenylbenzenes. These sulfated metabolites were confirmed as the ultimate electrophilic metabolites. 3) DNA addition, 1′-sulfooxyalkenylbenzenes can eliminate from sulfonate ion, and form an intermediate of carbocation. Carbocation subsequently covalently binds with adenine or guanine base to form DNA adducts, as depicted in Figure 5. Metabolic activation of the alkenylbenzenes to their ultimate carcinogens require the key catalysis of both CYP450s and sulfotransferases. In addition, reactive 1′-hydroxymyristicin showed chemical reactive activity which can react with NAC.

### 2.7 Metabolic Activation of Flavonoids-Induced Cytotoxicity

Flavonoids are natural phenolic acid components in the diet and medicinal plants. Dietary flavonoids are the most abundant secondary metabolites in the plant kingdom and they play a regulating or preventing action in many disorders or diseases for a long period of time. Currently, owing to their extensively biochemical and pharmacological effects, flavonoids obtain the burgeoning interest in complementary and alternative medicine.

**FIGURE 6:**
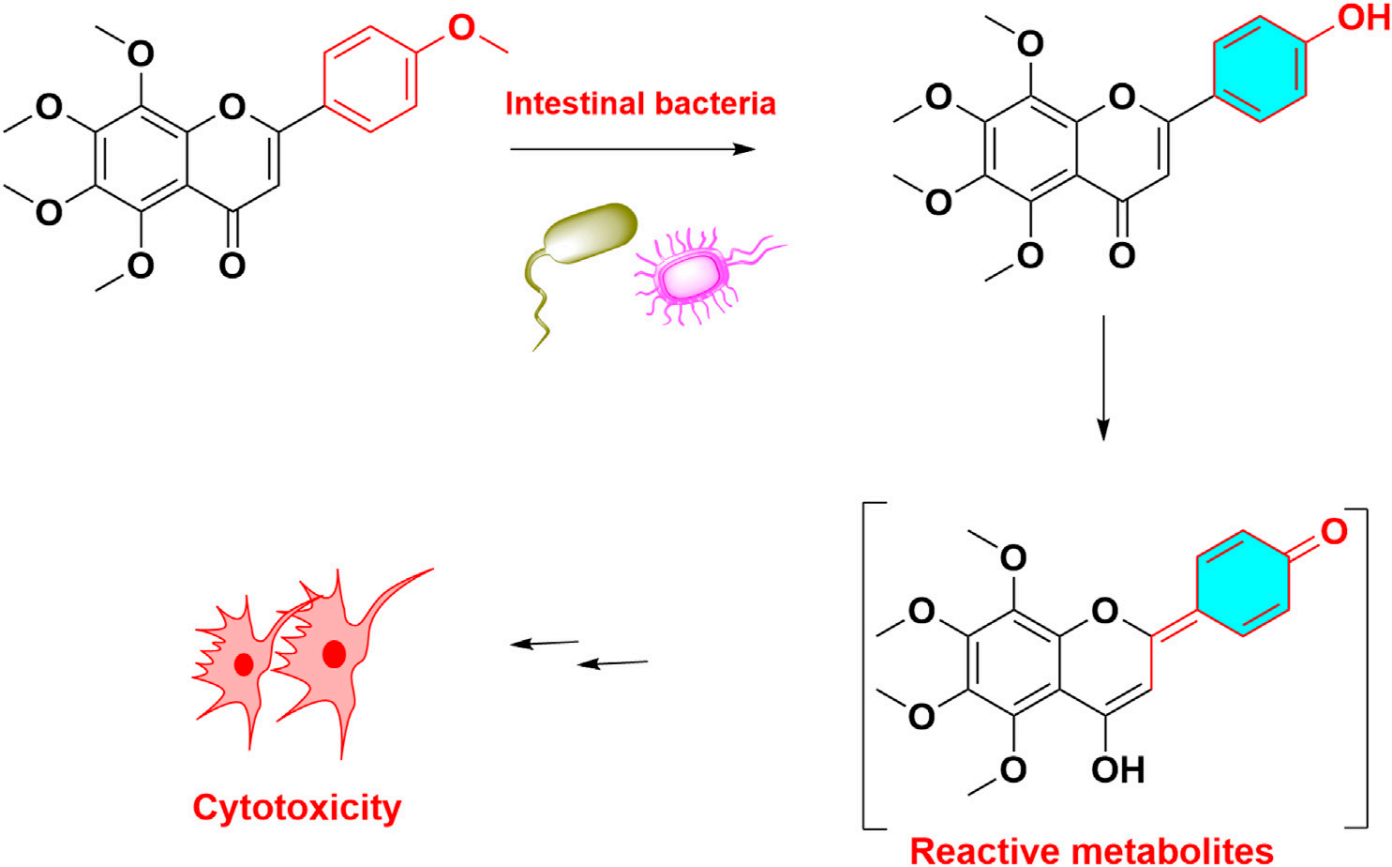
Metabolic activation and toxicity of tangeretin. Tangeretin demethylated and formed quinone methide, led to cytotoxicit.

Previous studies indicated that quercetin was mutagenic without microsomal activation (Bjeldanes and Chang, 1977). Recently, metabolic activation of quercetin majorly including initially enzymatic or chemical oxidation of quercetin, formation of quercetin ortho-quinone, followed by isomerisation of the ortho-quinone to quinone methides. These quinone methides were reported to be the alkylating DNA-reactive intermediates (Vrijsen et al., 1990). In addition to mutagenicity, multiple flavonoids can potentiate their cytotoxicity toward breast cancer cells after CYP1A1 and CYP1B1 mediated metabolic activation. And CYP1-mediated metabolic activation of dietary flavonoids enhanced their toxicity in breast cancer cells (Androutsopoulos et al., 2008; Androutsopoulos et al., 2009). Compared to other flavonoids, 4′-hydoxyl-flavonoids can easily transform to quinone methide, and showed higher cytotoxicity in Figure 6. It was reported that metabolic activation of numerous other flavonoids was involved in or aggravated their toxicities (Table 1). Taken together, quinone intermediate including quinone methides and ortho‐benzoquinone, were usually formed in the metabolism of most pre-toxic flavonoids, which can further result in the depletion of cellular GSH and DNA damage.

## 3 Intestinal Flora Mediated Metabolic Activation of Natural Products Leading to Toxicity

Gut flora extensively implicates in the metabolism of multiple medicinal drugs, consequences for interpersonal variation in drug or xenobiotics toxicity (Zimmermann et al., 2019; Wilson and Nicholson, 2017; Zheng et al., 2013; Alexander et al., 2017). Dietary natural products can be extensively metabolized in the gut, not only by digestive and intestinal mucosal enzymes, but also by the gut microbiota. Microbiome-driven drug metabolism can lead to adverse consequences and toxicity. Similar to CYP450s mediated metabolic activation, the intestinal microflora also can form reactive metabolites and significantly aggravate xenobiotic-induced toxicities via metabolic activation (Table 1). Additionally, the gut microbiota can affect drug metabolism and toxicity indirectly, such as competition of bacterial-derived metabolites for xenobiotic metabolism pathways or the modulation of host metabolic systems (Wilson et al., 2015).

Gut microbiota involves in many metabolic reactions, such as demethylation, dehydroxylation, deacylation, decarboxylation, and hydrolysis reactions as well as acetylation (Wilson et al., 2015). Hydrolysis products of glucosides can form their aglycones in the intestine, which are easily absorbed into blood, and circulate throughout the human body. In particular, intestinal bacterial secretes β-glucuronidase, which hydrolyzes glucuronidated metabolites to their toxic aglycones in intestines and results in intestinal damage. For example, intestinal microbiota-mediated geniposide bio-transform to genipin dialdehyde intermediate, leading to hepatotoxicity in rats (Li et al., 2019b). Pre-toxic arbutin can be hydrolyzed into toxic hydroquinone with the aid of intestinal flora transformation (Kang et al., 2011).

## 4 Metabolic Bioactivation of Natural Products Leading to Mechanism-Based Inactivation of CYP450s

CYP450s are the most common phase I metabolic enzymes, and involve in the majority of the metabolism of clinical drugs and natural products. Inhibition of CYP450s is by far the most common factor leading to drug-drug interaction (DDI). CYP450s inhibition can be classed as reversible (competitive or non-competitive) or irreversible (mechanism-based inactivation). In particular, mechanism-based inactivation (MBI) often involves metabolic bioactivation of natural products by drug metabolizing enzyme to an electrophilic reactive intermediate, which covalently modifies an active site amino acid residue and/or coordinates to the heme prosthetic group, and leads to quasi-irreversible or irreversible inactivation (Appiah-Opong et al., 2009; Kamel and Harriman, 2013). Compared to reversible inhibition, irreversible inhibition more frequently results in unfavorable DDIs as the inactivated P450 enzyme has to be replaced by newly synthesized protein. Natural flavonoids, phenylpropanoids, terpenoids, quinones, and alkaloids are mechanisms -based inactivators, may trigger herb-drug or food-drug interactions. thiophene, furan, alkylamines are common latent functional groups responsible for reactive metabolites induced MBI (Mirzaei et al., 2021). The risks for intake of naturally occurring irreversible P450 enzyme inhibitors have been rising, owing to the rapid growth of the global consumption of natural products (Zhang et al., 2020b). Mechanism-based inactivation, the structure of reactive metabolites−MBI relationships, should be applied in clinical to mitigate the risk of idiosyncratic drug toxicity (Orr et al., 2012).

## 5 Novel Analytical Techniques Used for Detection of the Reactive Metabolites of Natural Products

Metabolic activation of a natural product resulting in reactive metabolite(s) that can covalently modify proteins is considered an initial step that may lead to drug-induced organ toxicities, therefore, detecting and characterizing of reactive metabolites will provide a useful clue for predicting metabolic activation mediated toxicity. Ultra-high liquid chromatography coupled with mass spectrometry (UPLC‐MS) plays a key role as the predominant analytical platform for analysis and detection of reactive metabolites. However, it is difficult to detect most reactive metabolites directly. Reactive metabolites are liable to form stable adducts by covalent combination with trapping reagents, reactive metabolites related adducts make the reactive metabolites detectable. Trapping assays, especially glutathione trapping, are usually performed to detect reactive metabolites that can contribute to drug toxicity. These trapping reactions are often performed in liver microsomes with NADPH and appropriate nucleophilic trapping agents, such as thiols (glutathione (GSH), its ethyl ester derivative, or N-acetylcysteine), amines (semicarbazide and methoxylamine), or cyanide anion. In the UPLC–MS/MS, a neutral loss scan or a precursor ion scan mode are usually applied to detect of GSH-trapped reactive metabolites, however, the sensitivity and selectivity in the scan mode are sometimes poor owing to the interference of endogenous biological matrices derived from HLM incubation. In addition, the selectivity of GSH adduct in conventional reversed-C18 phase liquid phase separations is not sufficient, and needs to be improved to minimize false positive and/or negative results. It remains a challenge to analyze these reactive metabolites adducts. Glutathione labeled with a fluorescence tag of dansyl (dGSH) can be applied as a trapping agent for the fluorescent quantification and identification of hard reactive metabolites (Gan et al., 2005; Nishijo et al., 2020).On one hand, dGSH can increase the detection sensitivity of trapped reactive metabolites; On the other hand, dGSH captures only soft electrophilic reactive metabolites. CysGlu-Dan labeled cystine have been built to detect soft and hard electrophilic reactive metabolites (Shibazaki et al., 2021). Thus, a high‐throughput sensitive and selective GSH trapping assay using the combination of stable isotope‐labeled GSH and UPLC-MS system for identification and characterization of reactive metabolite “all‐in‐one” is recommended.

Metabolomics-based toxicology can evaluate toxicity and identify toxicological biomarker of natural product, which is helpful to guide clinical medication and reduce adverse drug reactions. UPLC-MS-based metabolomic approach is a sensitive, effective and unbiased tool for profiling of drug metabolism and metabolic activation (Li et al., 2011; Li et al., 2012; Hanhineva et al., 2017) to eliminate complex matrix interferences. It was reported that natural cocaine can form reactive metabolites, leading to hepatotoxicity. These reactive metabolites of cocaine were easily screened and analyzed by metabolomics (Yao et al., 2013). In addition, reactive metabolites-proteins adducts are thought to be a principal factor in natural drug-induced liver injury. targeted proteomics approach to the identification of peptides modified by reactive metabolites, which is generally suitable for the identification and characterization of modified proteins and metabolite structures involved in covalent binding and may serve as a valuable tool to link protein targets with clinically relevant toxicities (Tzouros and Pähler, 2009). Quantitative chemical proteomic profiled the *in vivo* toxic targets of reactive drug metabolites (Whitby et al., 2017).

## 6 Concluding and Perspective

The review provides a reference for the reasonable and safe usage of herbal and dietary natural products. In recent years, the toxicity of natural drugs has attracted widespread concerns around the world, especially for inappropriate dosage or overdose use. The toxicity of natural drugs largely affected their applications clinically. Metabolic activation of natural products can initiate or aggravate their hepatotoxicity, nephrotoxicity, and pulmonary toxicities.

Although some toxic components were discovered from natural herbal and dietary, there are still many unknown toxic compounds to be identified. Firstly, rapid high throughput approach screening and assessing the toxic component in natural drugs should be established. Considering that complex biological factors can affect the evaluation of metabolic activation leading to toxicity, the sensitive and specific method, UPLC-MS/MS, should be developed for exclusively detecting reactive metabolites and their GSH conjugates. Moreover, metabolic activation of many different natural products formats chemically reactive/toxic metabolites, that can result in toxicity through binding to macromolecular targets (proteins or DNA). Reactive metabolites -protein adducts can be selected as toxic marker for predicting and evaluating metabolic activation leading to toxicity, and nontargeted identification of reactive metabolite protein adducts is desirable. Methods for qualitative and quantitative detection of reactive metabolites-protein adducts should be developed and applied for the clinical diagnosis of toxic natural products exposure and toxic natural products-induced liver injury. The determination of reactive metabolites-protein adducts using mass spectrometry is an emerging area which allows comprehensive understanding of the underlying mechanisms involved in toxicity and reveal potential biomarkers of exposure or toxic response. More specific fluorescent probes for analysis and detection reactive metabolites should be designed for diagnosis the toxicities of natural products. Standard drug discovery and development strategies should be applied to natural products.

Although the molecular machinery underlying toxicity remains largely unclear, more toxic action should be clarified from the perspective of molecular biology or systematic biology in further study. It is essential to establish the diagnostic strategies to detect drug-induced toxicities clinically, which is also very important to find the strategy of detoxification to decrease the toxicity of natural drugs, and more comprehensive understandings of toxicity are urgently required.

Considering the multitude of potentially toxic natural products in multi-ingredient supplements, the unknown concentrations, and missing or inappropriate labels, as well as the absorption, distribution, metabolism and excretion in the host, the varying phenotypic presentation and unpredictable spectrum of toxicity, making the diagnosis challenging, these above unaddressed issues warrant further study. The current toxic raw data of most natural drugs and products are too limited to utilize. Therefore, the toxic database for herbal and dietary natural products should be built to record the side/toxic effects of natural drugs and their clinical formulation, and determine toxic components for the further investigation. The known potentially toxic indigents and their toxic effects should be labeled on the package of herbal and dietary supplements.
